# Evaluating the impact of digitalization, renewable energy use, and technological innovation on load capacity factor in G8 nations

**DOI:** 10.1038/s41598-023-36373-0

**Published:** 2023-06-05

**Authors:** Usman Mehmood, Salman Tariq, Muhammad Umar Aslam, Ephraim Bonah Agyekum, Solomon Eghosa Uhunamure, Karabo Shale, Mustafa Kamal, Muhammad Faisal Khan

**Affiliations:** 1 Remote Sensing GIS and Climatic Research Lab National Center of GIS and Space Applications, University of the Punjab, Lahore, Pakistan; 2 Department of Political Science, University of Management and Technology, Lahore, Pakistan; 3 Department of Space Science, University of the Punjab, Lahore, Pakistan; 4 Department of Nuclear and Renewable Energy, Ural Federal University Named After the First President of Russia Boris Yeltsin, 19 Mira Street, Ekaterinburg, 620002 Russia; 5 Faculty of Applied Sciences, Cape Peninsula University of Technology, P. O. Box 652, Cape Town, 8000 South Africa; 6 Department of Basic Sciences, College of Science and Theoretical Studies, Saudi Electronic University, Dammam, 32256 Saudi Arabia; 7 Department of Basic Sciences, College of Science and Theoretical Studies, Saudi Electronic University, Riyadh, 11673 Saudi Arabia

**Keywords:** Climate sciences, Ecology, Environmental sciences, Energy science and technology

## Abstract

Ecosystems are in danger due to human-caused air, water, and soil pollution, so it is important to find the underlying causes of this issue and develop practical solutions. This study adds to environmental research gap by suggesting the load capability factor (LCF) and using it to look at the factors affectting environmental health. The load capacity factor simplifies monitoring environmental health by illustrating the distinction between ecological footprint and biocapacity. We examine the interplay between mobile phone users (Digitalization DIG), technological advancements (TEC), renewable energy use, economic growth, and financial development. This study assesses G8 economies’ data from 1990 to 2018, using a Cross-Section Improved Autoregressive Distributed Lag CS-ARDL estimator and a cointegration test. The data shows that green energy, TEC innovation, and DIG are all beneficial for natural health. Based on the results of this study, the G8 governments should focus on environmental policies that promote economic growth, increase the use of renewable energy sources, guide technological progress in key areas, and encourage the development of digital information and communications technologies that are better for the environment.

## Introduction

Sustainable development is a big topic in the thoughts of scientists, governments, and international organizations. Every nation strives for sustainable development that fulfils the current generation's expectations without compromising future generations potential to satisfy their needs. In 1987, the commission of Brutland defined sustainable development^1–3^. In achieving sustainable development gains, nations must tackle issues that include the spread of contagious diseases, global warming, the discharge of greenhouse gases (primarily carbon (CO_2_), and the usage of environmentally unfriendly fuels^4^. The Intergovernmental Panel on Climate Change (IPCC) reported that the increasing worldwide carbon dioxide emissions had been the primary cause of climate change since 1750. An increase of 52% in greenhouse gas emissions is expected by 2050 if nations do not enact effective climate change laws^5^. Experts are investigating the root causes of environmental deterioration within the framework of sustainable development. Economic development is a crucial factor in shaping our natural world. As stated by Ref.^6^, environmental deterioration increases but reduces when per capita income rises over a particular threshold. How does the per capita income impact the environmental transformation mechanism work? Agriculture and heavy industries in developing nations are significant sources of pollution because they use outdated methods and equipment. Cleaner industrial techniques made possible by this shift help lower pollution. Using a wide variety of environmental indicators, several researchers keep digging further into the EKC hypothesis^7^. CO_2_ may be the utmost used independent variable; however, there is yet to be a consensus among theoretical experts on which dependent variable should be used. Researchers used greenhouse gas emissions (both direct and indirect) and biodiversity loss as dependent variables, and others have deforestation, urban wastes, and water quality^8^. However, these factors only provide a partial aspect of the environmental issue when used in isolation. Calculating the EKC theory without considering the reduction in other pollutants while focusing entirely on the rise in CO_2_ emissions might be misleading^9^. Air, water, and land pollution are all environmental problems that should be considered when examining a country's capacity to maintain its ecological sustainability in the long run.

In comparison, the EF represents human needs for natural resources and environmental deterioration without considering ecological reactions to these demand or supply opportunities. When calculating an organization’s ecological footprint, the EF is regarded as the supply side, whereas biocapacity is the demand side. A more all-encompassing measure, such as renewing biocapacity, is required for a more accurate sustainability assessment^9^. Biocapacity is essential to human existence, and failing to account for it in environmental assessments leads to inaccurate outcomes^10^. The Load Capacity Factor (LCF), considered biocapacity (EF), has been suggested by Ref.^11^ to conduct more accurate environmental impact assessments. In light of this suggestion, Ref.^12^ performed the first empirical investigation of the determinants of LCF using a linear model to investigate the connection. Capital gains and income are two different things. As the EKC theory proposes, income and LCF may not have a straight-line relationship. Due to the LCF’s role as an indicator of environmental quality, the negative link between the environment and income is significant. It is clear that the LCF drops during the beginning of economic development but then rises after income reaches a specific threshold. The LCF hypothesis describes the relationship between load and capacity. According to the LCF theory, a country may reduce its EF while growing its biocapacity after achieving a certain revenue level. We can look at environmental deterioration and quality by utilizing LCF. For a booming economy, a pristine natural environment, societal advantages, renewable energy groups, consumption, and technical innovation are essential^13^.

By the year 2050, if capacity increases are combined with hydropower expansion, 3 billion metric tons (t) of carbon dioxide (CO_2_) emissions might be avoided. Renewable energy can boost the economy and social standing of outlying rural areas. Achieving this objective relies heavily on developing and maintaining renewable energy infrastructure in rural and remote areas, where energy access is more challenging (and millions still need more power)^12^. One of the most excellent methods to protect the environment is to switch to renewable energy^14^. Solar energy leaves no environmental trace and no waste, wind energy is also clean^15^. Instead of harming the local community, renewable energy sources like solar, wind, and other forms of natural biomass energies have become increasingly popular. These types of renewable energy with these features can boost the LCF and aid in realizing the SDGs. Moreover, the ecosystem consists of investments in research and development (TEC), which encourage the creation of novel goods, procedures, and information^16^. Through sponsoring research and development and creative ideas, it may be possible to stimulate the use of cleaner manufacturing processes, such as renewable energy and environmentally friendly technologies^17^. Spending more on research and development may be one way for businesses to realize their clean manufacturing goals^18^.

**Figure 1:**
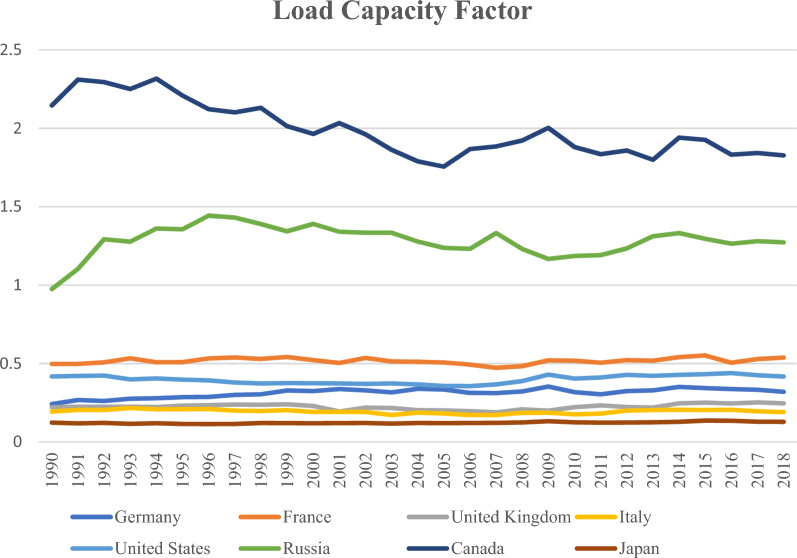
LCF trend; (Global footprint network).

Significant investments in research and development are required to overcome significant environmental concerns such as reliance on fossil fuels, high energy prices, an insufficient supply of renewable energy, and uncertainty about future energy sources' sustainability and reliability. As a result, investing in research and development has the potential to simultaneously drive the expansion of the economy and mitigation of environmental degradation. Compared to other aspects of the environment, such as expenditure on exploring alternative energy sources, information, and communications technology (DIG) is a comparatively younger development. The impact of technology on people's day-to-day lives is growing at a rate that is directly correlated to the pace at which technological advancements are made. Since the 1990s, people have used “information and communications technology”, abbreviated as “DIG”. The impacts of DIG can be seen in every aspect of contemporary life^19^. It is because technology is a driving force behind the SDGs’ social, economic, and environmental precepts, DIG is essential to the objectives^20^. For example, the extensive usage of the internet^21^, the fall of paper consumption, and the development of telephone and videoconferencing that allows people to work remotely all contribute to environmental improvements^22^. DIG can optimize energy usage through digital media, telecommuting models, alternative consumption patterns, and digital process management and help lower resource consumption^23^. Improved information and communication technology (DIG) infrastructure benefits a country's economy's private and public sectors^24^. The G8 nations have improved output, economic efficiency, social change, and technical advances through increased investment and digitized business ventures^25^. Despite their reputation as a top-tier, high-income industrial powerhouse, not all G8 countries are exceptional in environmental performance^26^. Figure 1 shows that all G8 nations, except Canada and Russia, are below the Limit of Sustainability. After avoiding a decline between 1990 and 2018, the LCF levels in Germany, France, and the United States are still well below “1”. It is, therefore, essential to look into the environmental issues faced and solutions implemented by the G8 nations^20^.

As a continuation, this research focuses on the G8 countries for the following reasons: G8 nations have expand its economies by creating the way for rapid advances in commerce and industry. Still, their efforts to do so come at the expense of environmental quality^27^; secondly, because environmental deterioration is so severe, officials in the G8 countries must look into the causes of environmental degradation; however, previous empirical work has been largely focused on ecological destruction measures resulting from human consumption such as EF^28,29^. The EF collects data on air, water, and soil pollution, but the extent to which they deplete these supplies needs to be made clear. This research helps bridge the gap by proposing a novel ecological economics hypothesis^30^. The cross-sectional dependency in the data, the CS-ARDL, and Durbin-Hausmann cointegration techniques are applied (CSD). Given the higher potential for the G8 nations to influence one another, the findings of CSD research may be instructive. Figure 1 shows the LCF of G8 countries during 1990–2018. This study contributes to knowledge gap about LCF (load capacity factor) in G-8 countries in light of technological advancement and renewable energy in the scenario of globalization. Preceding this study, some studies found that new technology, digitalization, and renewable energy impact LCF. Many studies have been done on LCF and other related variables in other countries or regions. This study contributes originality in the case of the G-8 economies.

This research has insightful data from various perspectives. This is how the investigation is presented: the literature on the effect of (EF) research and development, DIG, and renewable energy on the environment is discussed in Section “Review of the literature”. Studies investigating what factors influence the LCF are also discussed here. We described the data used and the research techniques employed in Section “Data and methodology”. Empirical findings are given and discussed in Section “Results and discussion”. The report concludes with several suggestions for future policy in Section “Conclusion and policy implications”.

## Review of the literature

There are four subsections in this section. The first part of this paper focuses on the connection between renewable energy and the natural world. In contrast, the subsequent parts examine the relationships between (TA) spending and the natural world. A synthesis of research into the LCF is provided, and research gaps are highlighted in the final section.

### The correlation between RE and the environment

From an environmental perspective, several studies have shown that renewable energy sources are preferable to fossil fuel. Two studies^31,32^, indicated that RE reduces EF in comparable high-polluting countries. The results of this research, however, were different from one another. However, as Ref.^9^ pointed out, the pace at which CO_2_ emissions rise while switching to renewable energy is only half that when switching to fossil fuels. In contrast, studies undertaken in Turkey^33^, and China^19^, concluded that the use of renewable sources of energy does not significantly reduce CO_2_, EF, or LCF and that there is no consumption of renewable resources responsibly or efficiently^10^. In a study by Mehmood et al.^34^ estimated the impact of the transition to green energy, economic growth, and natural resource availability on carbon dioxide emissions in the context of evolving political, economic, and financial risks. The findings indicate that using natural resource rents is positively associated with CO_2_ emissions, whereas adopting green energy sources is negatively correlated with CO_2_ emissions. Mitigating political and financial risks can lead to reduced carbon dioxide emissions and improved environmental conditions. Furthermore, it has been observed that the restriction of CO_2_ emissions occurs after a certain threshold of economic growth has been achieved, thereby validating the existence of an inverted U-shaped relationship between economic growth and CO_2_.

Hence, recognizing the necessity for a more discerning assessment of the complexities entailed in investigating the ecological footprint, this research scrutinizes the interconnections among natural resources, technological advancements, financial expansion, and the consequent ecological footprint in developing nations^35^.

### The relationship between TEC spending and the environment

TEC costs cut the EF in all 28 EU member states, according to Ref.^36^. The authors of Ref.^37^ found that supporting research and development reduces carbon dioxide emissions using CCEMG and AMG panel data estimators. Similarly, Ref.^38^ utilized Tobit and probity models to evaluate Chinese firm data and discovered that increasing TEC spending would enhance green innovation efforts and success. Fareed et al. (2022) found in panel quantile regression research of Ref.^38^ European Union states that expenditure on TEC minimizes the bad impacts of economic inclusion on environmental factors (EF), reducing or postponing ecological deprivation. An interactive fixed effect approach study of 27 nations undertaken by Ref.^39^ found that expenditure on renewable energy technological advancement (TEC) correlates with decreased (CH_4_) methane, (CO), (NO), and (CO_2_) carbon dioxide emissions^40^, using the Degree of Operating Leverage analysis, increasing investment in TEC reduced CO_2_ emissions in Saudi Arabia. Using Westerlund cointegration and multiple panel estimators, Ref.^4^ determined that TEC investment considerably and positively contributed to cutting GHG emissions across 40 nations^41^. Numerous reports have shown the environmental benefits of investing in research and development. Contrary to popular opinion, TEC investment causes environmental damage in 96 nations, as Ref.^42^ show using an inclusive nested spatial model. In a similar vein, Ref.^43^ found that TEC investment increases (EF) and pollution in five developing countries using the Panel Generalized Method of Moments and the Fully Modified Ordinary Least Squares.

### Relationship between DIG and the environment

Due to technology significance to progress, research into the impact of DIGs on pollution is ongoing. At the same time, the green benefit of DIGs is generally modest in developing nations; Ref.^44^ Gaussian Mixture Models examinations of 60 countries indicated that DIG adaptation in metropolitan zones helps cut CO_2_ emissions. Using the panel Vector Autoregressive Model methodology, Ref.^45^ discovered that DIG helps with CO_2_ reduction in 18 African countries by allowing for greater access to resources and carbon monitoring^46^. Finds that widespread usage of DIG has helped Chinese cities lower carbon intensity after considering geographical and temporal consequences. Using the (CUP-FM) estimator^47^, discovered that DIG reduces (CO_2_) emissions in 17 countries in Asia. According to the authors, policymakers should prioritize environmental protection when formulating new legislation, and DIG-enabled economic policies should play a pivotal part in this effort. The influence of (DIG) on organizational effectiveness (EF) has been the focus of numerous studies utilizing various econometric approaches and used the ARDL panel to determine that DIG lessens (EF) in the world's top ten polluting countries. Kahouli et al.^48^ used Johansen cointegration and (VECM) to discover that DIG reduces (EF) in Saudi Arabia. A study using the AMG estimator^49^ found that internet usage was associated with decreased EF across all G8 countries. Studies consistently show that information and communication technologies are harmful to the environment. The G8 has been demonstrated to have lower CO_2_ emissions due to the usage of DIG; however, is found the reverse to be true when using PMG. Analysis of data from the N11 nations conducted by^50^ using the Feasible General Least Squares (FGLS) technique indicated that DIG raises the EF. The study of Ref.^51^ utilized PMG to get the same EF results for the BRICS countries. To investigate the influence of DIG on EF. Reference^43^ analyzed 47 nations in Sub-Saharan Africa using the FGLS and PCSE. Kazemzadeh et al.^52^ obtained similar results from panel quantile regression analysis performed on 19 developing countries, concluding that DIG does not significantly affect EF.

### LCF empirical research

Empirical research has usually concentrated on the part that CO_2_ emissions and the EF play in these problems; however, much research has been done on the causes of climate change. The ARDL approach, modified for the United States and Japan, allowed the simultaneous investigation of biocapacity and EF to investigate the variables that influence LCF. This groundbreaking study showed that LCF is restrained by financial resources in the United States. Used the dynamic ARDL approach to determine that in China, energy intensity, resource rent, and income negatively influence LCF, but human capital has a favorable effect. Specialists have studied the factors that impact LCF in the subject. The authors of^53^ argue that although applying the (ARDL) method to India and income does reduce LCF, all contribute to better environmental results. Awosusi et al.^54^ used ARDL for Brazil and found that globalization increases environmental quality, but urbanization has little effect on LCF. The literature review is divided into four parts, each addressing a different area of study that needs addressing. First, research on the causes of LCF in the Group of Seven needs to be completed.

Furthermore, there needs to be more studies examining how DIG affects LCF. Does information and communication technology affect biological potential? Do increased Internet access, mobile phone use, and other IT developments positively or negatively affect the world's natural resource supply? By addressing these issues, we may contribute new knowledge and close gaps in the existing body of research^55^.

In addition, developments in the building industry in G20 countries worsen environmental quality. Finally, earlier studies show that technological progress has only sometimes had beneficial effects on environmental quality. Empirical studies have shown conflicting results. Thus, additional research into its impact on environmental quality is required. There is also a need for more information on how BRICS-T country green tech innovations have affected ecological quality. Therefore, the BRICS-T nations are the primary focus of this study.

This research aims to shed light on this problem and give a thorough environmental evaluation of OECD nations by analyzing the impact of human capital, income, natural resources, urbanization, and renewable energy on the load capacity factor for 26 OECD countries between 1980 and 2018. This research tests the load capacity curve (LCC) hypothesis using the recently established quantile common correlated effects mean group (QMG) estimator. Our findings provide credence to the LCC hypothesis and support a U-shaped relationship between wealth and environmental quality. Human capital, resource rent, and renewable energy all boost the load capacity factor, according to the QMG estimator, but urbanization hurts ecological quality. Table 1 shows the data symbols and origins used for the study.

**Table 1:** Data symbols and origins.

Indicators	Sign	Calculating unit	Source
Load capacity factor	LCF	Biocapacity/ecological footprints	GFN^56^
Digitalization	DIG	Fixed broadband subscription (per 100 persons)	GINI index^57^
Natural resources	NAT	Natural resources rent % of GDP	WDI^58^
Government stability	GOV	Governmental stability	ICRG^59^
Economic growth	GDP	Constant 2010 US $ (per capita)	WDI^58^
Financial development	FD	Domestic credit to the private sector	WDI^58^

In Table 1, the data for Load capacity factor (LCF) has been obtained from Global Footwork Network (GFN). The data for Digitalization has been derived from GINI index, while data for Natural Resources and Economic growth has been derived from World Data Indicators, (WDI). The data for Government stability has been derived from International country risk guide, (ICRG).

## Data and methodology

Before running a unit root test, it's important to rule out the possibility of cross-sectional dependence (CD). CD shares traits with economic integration, residual reliance, and shared stocks^60^. Neglecting (CD) can lead to skewed statistics, misaligned proportions, prejudiced stationarity, and skewed cointegration^61^. We used the Chudik and Pesaran^62^ CD test to identify potential problems. Once results from CD have been obtained, the panel data is examined with a unit root or stationarity technique.

### Cointegration testing

Next, after utilizing the unit root test, we apply an improved type of^63^ to check for slope homogeneity and heterogeneity. In CD, the attributes of size distortion in panel data cannot be predicted by the first generation of cointegration tests established and used previously^64^. None of the researchers took CD in a cross-sectional study into account. The approach developed versions for slope, CD, and correlated error variation.

### CS-ARDL

This research used the CS-ARDL method to calculate the short- and long-term coefficients. As previously mentioned^65^, CS-ARDL employs a DCCI (dynamic common correlated impact) predictor to address the issues of heterogeneity and cross-section dependence. Equation (1) is the mathematical representation of CS-ARDL.

1 $${H}_{i,t}= \sum_{I=0}^{{p}_{w}}{\gamma }_{I,i}{W}_{i,t-1}+\sum_{I=0}^{{p}_{z}}{\beta }_{I,i}{Z}_{i,t-I}+{\varepsilon }_{i,t}$$

In the presence of CD, using Eq. (1), based on the autoregressive distributed lag (ARDL) model, will lead to indeterminate results. Regressor variables are averaged over cross-sections to alter Eq. (2). As a result, and we can rest any doubts about the existence of the CD-induced threshold effect^62^.

2 $${H}_{it}= \sum_{I=0}^{{a}_{w}}{\gamma }_{I,i}{,H}_{i,t-1}+\sum_{I=0}^{{a}_{z}}{\beta }_{I,i}{Z}_{i.t-I}+\sum_{I=0}^{{a}_{x}}{{\alpha }^{^{\prime}}}_{i},I{\overline{X} }_{t-I}+{\varepsilon }_{i,t}$$

whereas,

$${\overline{X} }_{t-I} = {\overline{H} }_{i,t-I}{\overline{Z} }_{i,t-I}$$

Existing delays among all parameters are denoted by aw, as, and axe. The independent variables are represented by Z (i.t), while the dependent variable, H, is the per capita carbon emission based on consumption. The average cross-sectional value (X) is used instead of considering trends to counteract the spillover mentioned above.

## Results and discussion

First, we check for (CSD), between countries. Cross-sectional dependence (CSD) measures how one country’s economic fortunes affect those of another. Traditional panel data estimators may need more reliability and efficiency if independent variables cause the CSD. Parameter inconsistency is another result of CSD^66^. Additionally, series with CSD fail conventional unit root testing^67^. To begin, we employ the CD test of^68^ and the (LM) test of (Breusch and Pagan) to examine whether or not CSD exists. Table 2 presents the outcome of the (CSD) analysis. We found evidence against the absence of CSD, as indicated by the CD and LM tests.

**Table 2:** CD test.

Variable	Test statistics
LCF	27.351^a^
RE	25.605^a^
DIG	24.532^a^
TEC	21.372^a^
GDP	28.494^a^
FD	28.418^a^

^a^Explains the level of significance at 1%.

The outcomes of the Pesaran CIPS unit root test^69^ are shown in Table 3. No discernible pattern in GDP or DIG growth was found (0). The (LCF), (RE), and TEC all have a unit root, but the initial disparities between them and the mean are stationary (1). Assuming that the independent variables are integrated at distinct orders (I (0) and I (1)), here we also discover that the dependent variable is blended at I (1). Since the Westerlund cointegration test (see Table 4) permits the investigation of long-run correlations between variables of varying integration orders, we use it. Adding CSD to the display is another advantage of this test.

**Table 3:** Unit root test.

Variable	CADF test		CIPS	
	At level	1st difference	At level	1st difference
${lnLCF}_{t}$	− 2.286	− 3.732^a^	− 1.722	− 3.796^a^
${lnRE}_{t}$	− 1.684	− 2.924^a^	− 3.406	− 5.958^a^
${lnDIG}_{t}$	− 3.843	− 3.948^a^	− 2.431	− 4.054^a^
${lnTEC}_{t}$	− 2.895	− 3.566^a^	− 2.934	− 3.534^a^
${lnGDP}_{t}$	− 2.014	− 4.485^a^	− 1.651	− 4.332^a^
${lnFD}_{t}$	− 2.209	− 3.328^a^	− 2.160	− 3.946^a^

^a^Shows significance @ 1%.

**Table 4:** Westerlund test.

Stat	Value	Z value	P value
G_t_	− 3.045^a^	− 1.775	0.038
G_a_	− 8.957	− 1.476	0.930
P_t_	− 9.152^a^	− 2.839	0.002
P_a_	− 10.838	− 0.572	0.284

^a^Explains the level of significance at 1%.

In the next stage, we evaluate the short- and long-run determinants of the (LCF) using the (CS-ARDL) approach. Table 5 demonstrates the results of the (CS-ARDL) approach.

**Table 5:** CS-ARDL.

	Value	Send	Significance
Short run			
${\Delta lnRE}_{t}$	0.033^a^	0.001	0.000
${\Delta lnDIG}_{t}$	0.006^a^	0.002	0.000
${\Delta lnTEC}_{t}$	0.087^a^	0.001	0.000
${\Delta lnGDP}_{t}$	− 0.027^a^	0.003	0.001
${\Delta lnFD}_{t}$	0.054^a^	0.008	0.000
Long run			
${lnRE}_{t}$	0.078^a^	0.000	0.000
${lnDIG}_{t}$	0.033^a^	0.000	0.000
${lnTEC}_{t}$	0.046^a^	0.003	0.000
${lnGDP}_{t}$	− 0.014^a^	0.002	0.000
${lnFD}_{t}$	0.028^a^	0.000	0.000
ECM	− 0.451^a^	0.009	0.000

^a^Shows significance @ 1%.

The CS-ARDL estimator concludes that RE, DIG, TEC, and FD contribute to a better environment over time. A 1% rise in renewable energy sources and a 1% increase in TEC are both found to boost LCF by 0.078% and 0.046%, respectively. While DIG also boosts LCF by 0.033% and FD by 0.028% in G8 countries. On the other hand, GDP in G8 nations is negative by -0.014% and does not support LCF here. According to data collected from WDI (World Data Indicators), Ref.^11^ research on the United States and Japan^19^ and research on the G8 countries both find that renewable energy plays an important influence in Ecological Footprints. The advanced technologies and robust renewable energy infrastructures of the G8 nations help to protect the environment.

In contrast to Ref.^22^, we find that TEC has a positive environmental impact in the G8 countries, as^49^ also notes. Incorporating TEC's adaptability and environmentally friendly manufacturing technologies can lessen the environmental burden and boost the production of useful biomaterials. Therefore, the G8 nations can boost their environmental protection by bolstering their TEC systems and spreading green TEC practices.

The LCF is positively correlated with TEC spending, with a 1% rise in TEC spending leading to a 0.046 percentage point increase in the LCF. In contrast to their long-term benefits, the immediate advantages of green energy and TEC are greater^70^, found the same thing for the G8 countries, so this conclusion is consistent with their research. Increased investment in TEC by the G8 governments can greatly increase the LCF by stimulating the creation of innovative clean energy technologies, raising public consciousness about the importance of these issues, and improving energy efficiency. In contrast, FD is positively important by 0.028%. As a result, FD is considered to be ecologically benign in G8 nations.

This result also holds at the 5% significance level in the near run. The findings show that the ECM is negative, less than 1, and significantly statistically different from zero. Short-run imbalances are typically adjusted in 3.3 years, according to the ECM coefficient of 0.451. Table 6 presents the robustness check.

**Table 6:** Robustness check.

Variables	AMG	FMOLS
${lnRE}_{t}$	0.011^a^	0.062^a^
${lnDIG}_{t}$	0.013^b^	0.055^a^
${lnTEC}_{t}$	0.036^a^	0.030^a^
${lnGDP}_{t}$	− 0.063^a^	− 0.025^a^
${lnFD}_{t}$	0.072^a^	0.065^a^

^a^ and ^b^Explain the level of significance at 1% and 5% respectively.

To check the authenticity of the findings, this work applies AMG and FMOLS tests. These tests efficiently analyze the panel data and can accommodate the CD in the data. The findings show that RE, DIG, TEC, and FD are ecologically supportive, but economic growth degrades the environment. These results endorse the findings of CS-ARDL.

## Conclusion and policy implications

Several anthropogenic environmental problems pose hazards to human health and a country’s economy. Many scholars have used the environmental Kuznets curve to examine the causes of these issues. The environmental Kuznets curve employs indicators such as carbon dioxide emissions and ecological footprints, but more is needed to capture the complete scope of environmental problems. By utilizing cutting-edge second-generation panel data methods for the G8 countries, this research focuses on the load capacity factor within this context. This research seeks new insight into environmental economics by examining the effect of technological advancement, renewable energy, and digitalization on the load capacity factor. Our study offers crucial insight for G8 officials interested in enhancing environmental quality.

The cross sectional autoregressive distributed lag results demonstrate that green energy sources, government investment in technological advancement, and the adoption of cutting-edge information and communication technologies boost load capacity factor over time. So environmental strategies considering technological advancement, renewable energy, digitalization, and financial development are necessary to enhance environmental quality. As a first point, there is a noticeable link between financial development and load capacity factor (LCF). Growth in these economies leads to environmental problems like the overuse of natural fuels and other resources. To ensure environmental quality and protect nature, the governments of the four countries need additional policies and measures beyond economic growth. Second, the positive impact renewable energy has on the planet demonstrates the need for the G8 countries to increase their use of solar, wind, and biomass power and the proportion of their total energy usage from these renewable resources. Third, the savings from implementing digitalization in sectors like transportation, power, and communications free up capital for other environmentally beneficial projects. Using smart digitalization tools, the G8 nations can modernize their industrial structure in an ecologically responsible and efficient way regarding energy use. Green digitalization should also be incorporated into new forms of logistics service, carbon reduction technologies, and energy-saving procedures in the G8 nations. The load capacity factor can benefit greatly from all of these, and they can all help it grow. As a result, the G8 countries should prioritize technological advancement funding as a key component of their environmental policies and encourage the creation of more sustainable methods of manufacturing. Our research shows that technological advancement investments add more to rising load capacity factor than either renewable energy or digitalization. Fourth, our research findings offer crucial insights for policymakers in G8 nations seeking to enhance environmental quality. The findings obtained from the panel cointegration test indicate that the variables exhibit cointegration. The results obtained from the cross sectional autoregressive distributed lag (CS-ARDL) estimators indicate that the short and long-term LCF is positively affected by renewable energy consumption, technological advancement spending, and financial development. Fifth, to achieve this objective, governments may implement various energy policies, including renewable portfolio standards, clean energy subsidies, and tax exemptions for corporations that boost their renewable energy investment and consumption. So it is recommended that the G8 nations consider expanding their technological advancement and digitalization equipment.

Spending on TEC (technological advancement) by the G8 nations should prioritize innovations that improve energy efficiency, lower the price of energy, and make it easier to switch to renewable sources. Putting more money towards technological advancement initiatives is another way to assist the environment. In addition, technological advancement incentives can promote load capacity factor enhancement via less polluting industrial production methods, improved refuse recycling technology, and transportation activities that do not rely on fossil fuels. Finally, the novel hypothesis proposed in this work invites new lines of inquiry. In the future, researchers may choose to examine different sets of nations. Human capital, environmental taxes, financial development, and international trade are all potential load capacity factors that could be fascinating to investigate. Findings from recent studies will help us create a more thorough plan for ecological progress.

## Data Availability

All data generated or analyzed during this study are included in this published article.

## Abbreviations

EF: Ecological footprints
DIG: Digitalization
TEC: Technological advancement
RE: Renewable energy
GDP: Gross domestic product
FD: Financial development
LCF: Load capacity factor
CS-ARDL: Cross-section improved autoregressive distributed lag
EKC: Environmental Kuznets curve
PMG: Pooled mean group
NAT: Natural resources
GOV: Government stability

## References

[1] Liu H, Lei H, Zhou Y (2022). How does green trade affect the environment? Evidence from China. J. Econ. Anal..

[2] Meng Y, Liu L, Xu Z, Gong W, Yan G (2022). Research on the heterogeneity of green biased technology progress in Chinese industries—Decomposition index analysis based on the slacks-based measure integrating (SBM). J. Econ. Anal..

[3] Ren S, Liu Z, Zhanbayev R, Du M (2022). Does the internet development put pressure on energy-saving potential for environmental sustainability? Evidence from China. J. Econ. Anal..

[4] Shahzadi I, Yaseen MR, Iqbal Khan MT, Amjad Makhdum MS, Ali Q (2022). The nexus between research and development, renewable energy and environmental quality: Evidence from developed and developing countries. Renew. Energy.

[5] Grossman GM, Krueger AB (1991). Environmental impacts of a North American free trade agreement. NBER Working Pap..

[6] T. Panayotou. Empirical tests and policy analysis of environmental degradation at different stages of economic development. (1993).

[7] Zeraibi A (2022). Revisiting the EKC hypothesis by assessing the complementarities between fiscal, monetary, and environmental development policies in China. Environ. Sci. Pollut. Res..

[8] Uddin GA, Alam K, Gow J (2016). Does ecological footprint impede economic growth? An empirical analysis based on the environmental Kuznets curve hypothesis. Aust. Econ. Pap..

[9] Altıntaş H, Kassouri Y (2020). Is the environmental Kuznets Curve in Europe related to the per-capita ecological footprint or CO2 emissions?. Ecol. Ind..

[10] Pata UK, Balsalobre-Lorente D (2022). Exploring the impact of tourism and energy consumption on the load capacity factor in Turkey: A novel dynamic ARDL approach. Environ. Sci. Pollut. Res..

[11] Pata UK (2021). Do renewable energy and health expenditures improve load capacity factor in the USA and Japan? A new approach to environmental issues. Eur. J. Health Econ..

[12] Hu Y, Jiang W, Dong H, Majeed MT (2022). Transmission channels between financial efficiency and renewable energy consumption: Does environmental technology matter in high-polluting economies?. J. Clean. Prod..

[13] Mujtaba A, Jena PK, Bekun FV, Sahu PK (2022). Symmetric and asymmetric impact of economic growth, capital formation, renewable and non-renewable energy consumption on environment in OECD countries. Renew. Sustain. Energy Rev..

[14] Cui L, Weng S, Nadeem AM, Rafique MZ, Shahzad U (2022). Exploring the role of renewable energy, urbanization and structural change for environmental sustainability: Comparative analysis for practical implications. Renew. Energy.

[15] Chen C, Hu Y, Marimuthu K, Kumar PM (2021). Artificial intelligence on economic evaluation of energy efficiency and renewable energy technologies. Sustain. Energy Technol. Assess..

[16] Kahouli B (2018). The causality link between energy electricity consumption, CO2 emissions, R&D stocks and economic growth in Mediterranean countries (MCs). Energy.

[17] Luo S, Zhang S (2022). How R&D expenditure intermediate as a new determinants for low carbon energy transition in Belt and Road Initiative economies. Renew. Energy.

[18] Zafar MW, Shahbaz M, Hou F, Sinha A (2019). From nonrenewable to renewable energy and its impact on economic growth: The role of research & development expenditures in Asia-Pacific Economic Cooperation countries. J. Clean. Prod..

[19] Huang Y, Haseeb M, Usman M, Ozturk I (2022). Dynamic association between ICT, renewable energy, economic complexity and ecological footprint: Is there any difference between E-7 (developing) and G-7 (developed) countries?. Technol. Soc..

[20] Añón Higón D, Gholami R, Shirazi F (2017). ICT and environmental sustainability: A global perspective. Telemat. Inform..

[21] Dehghan Shabani Z, Shahnazi R (2019). Energy consumption, carbon dioxide emissions, information and communications technology, and gross domestic product in Iranian economic sectors: A panel causality analysis. Energy.

[22] Raheem ID, Tiwari AK, Balsalobre-Lorente D (2020). The role of ICT and financial development in CO2 emissions and economic growth. Environ. Sci. Pollut. Res..

[23] Wen H, Lee CC, Song Z (2021). Digitalization and environment: How does ICT affect enterprise environmental performance?. Environ. Sci. Pollut. Res..

[24] Yuan S (2021). Digitalization of economy is the key factor behind fourth industrial revolution: How G7 countries are overcoming with the financing issues?. Technol. Forecast. Soc. Chang..

[25] Ramzan M, Raza SA, Usman M, Sharma GD, Iqbal HA (2022). Environmental cost of non-renewable energy and economic progress: Do ICT and financial development mitigate some burden?. J. Clean. Prod..

[26] Belkhir L, Elmeligi A (2018). Assessing ICT global emissions footprint: Trends to 2040 & recommendations. J. Clean. Prod..

[27] Bekaroo G, Bokhoree C, Pattinson C (2016). Impacts of ICT on the natural ecosystem: A grassroot analysis for promoting socio-environmental sustainability. Renew. Sustain. Energy Rev..

[28] Pohl J, Frick V, Finkbeiner M, Santarius T (2022). Assessing the environmental performance of ICT-based services: Does user behaviour make all the difference?. Sustain. Prod. Consum..

[29] Nchake MA, Shuaibu M (2022). Investment in ICT infrastructure and inclusive growth in Africa. Sci. Afr..

[30] Ghosh S, Balsalobre-Lorente D, Doğan B, Paiano A, Talbi B (2022). Modelling an empirical framework of the implications of tourism and economic complexity on environmental sustainability in G7 economies. J. Clean. Prod..

[31] Khan AA (2022). Role of institutional quality and renewable energy consumption in achieving carbon neutrality: Case study of G-7 economies. Sci. Total Environ..

[32] Xie Q, Adebayo TS, Irfan M, Altuntaş M (2022). Race to environmental sustainability: Can renewable energy consumption and technological innovation sustain the strides for China?. Renew. Energy.

[33] Caglar AE, Mert M, Boluk G (2021). Testing the role of information and communication technologies and renewable energy consumption in ecological footprint quality: Evidence from world top 10 pollutant footprint countries. J. Clean. Prod..

[34] Ahmad M, Ahmed Z, Khan SA, Alvarado R (2023). Towards environmental sustainability in E−7 countries: Assessing the roles of natural resources, economic growth, country risk, and energy transition. Resour. Policy.

[35] Ahmad M (2020). The dynamic impact of natural resources, technological innovations and economic growth on ecological footprint: An advanced panel data estimation. Resour. Policy.

[36] Adedoyin FF, Alola AA, Bekun FV (2020). An assessment of environmental sustainability corridor: The role of economic expansion and research and development in EU countries. Sci. Total Environ..

[37] Petrović P, Lobanov MM (2020). The impact of R&D expenditures on CO2 emissions: Evidence from sixteen OECD countries. J. Clean. Prod..

[38] Zhang D, Jin Y (2021). R&D and environmentally induced innovation: Does financial constraint play a facilitating role?. Int. Rev. Financ. Anal..

[39] Khan AA (2022). Role of institutional quality and renewable energy consumption in achieving carbon neutrality: Case study of G-7 economies. Sci. Total Environ..

[40] Hailemariam A, Ivanovski K, Dzhumashev R (2022). Does R&D investment in renewable energy technologies reduce greenhouse gas emissions?. Appl. Energy.

[41] Omri A, Kahouli B, Afi H, Kahia M (2022). Environmental quality, healthcare and research and development in Saudi Arabia. Environ. Sci. Pollut. Res..

[42] Chen Y, Lee CC (2020). Does technological innovation reduce CO2 emissions? Cross-country evidence. J. Clean. Prod..

[43] Awad A (2022). Is there any impact from ICT on environmental quality in Africa? Evidence from second-generation panel techniques. Environ. Challeg..

[44] Chatti W, Majeed MT (2022). Information communication technology (ICT), smart urbanization, and environmental quality: Evidence from a panel of developing and developed economies. J. Clean. Prod..

[45] Shobande OA, Asongu SA (2022). The critical role of education and ICT in promoting environmental sustainability in Eastern and Southern Africa: A panel VAR approach. Technol. Forecast. Soc. Change.

[46] Sun H (2022). What are the roles of green technology innovation and ICT employment in lowering carbon intensity in China? A city-level analysis of the spatial effects. Resour. Conserv. Recycl..

[47] Zafar MW, Zaidi SAH, Mansoor S, Sinha A, Qin Q (2022). ICT and education as determinants of environmental quality: The role of financial development in selected Asian countries. Technol. Forecast. Soc. Change.

[48] Kahouli B, Hamdi B, Nafla A, Chabaane N (2022). Investigating the relationship between ICT, green energy, total factor productivity, and ecological footprint: Empirical evidence from Saudi Arabia. Energ. Strat. Rev..

[49] Özpolat A (2022). How does internet use affect ecological footprint?: An empirical analysis for G7 countries. Environ. Dev. Sustain..

[50] Kongbuamai N, Bui Q, Adedoyin FF, Bekun FV (2022). Developing environmental policy framework for sustainable development in Next-11 countries: The impacts of information and communication technology and urbanization on the ecological footprint. Environ. Dev. Sustain..

[51] Rout SK, Gupta M, Sahoo M (2022). The role of technological innovation and diffusion, energy consumption and financial development in affecting ecological footprint in BRICS: An empirical analysis. Environ. Sci. Pollut. Res..

[52] Kazemzadeh E, Fuinhas JA, Salehnia N, Osmani F (2022). The effect of economic complexity, fertility rate, and information and communication technology on ecological footprint in the emerging economies: A two-step stirpat model and panel quantile regression. Qual. Quant..

[53] Akadiri SS, Adebayo TS, Riti JS, Awosusi AA, Inusa EM (2022). The effect of financial globalization and natural resource rent on load capacity factor in India: An analysis using the dual adjustment approach. Environ. Sci. Pollut. Res..

[54] Awosusi AA (2022). A roadmap toward achieving sustainable environment: Evaluating the impact of technological innovation and globalization on load capacity factor. Int. J. Environ. Res. Public Health.

[55] Xu D (2022). Load capacity factor and financial globalization in Brazil: The role of renewable energy and urbanization. Front. Environ. Sci..

[56] Home - Global Footprint Network. https://www.footprintnetwork.org/.

[57] Solt F (2016). The standardized world income inequality database*. Soc. Sci. Q..

[58] World Bank Open Data. *World Bank Open Data* https://data.worldbank.org.

[59] The International Country Risk Guide (ICRG) – PRS Group. https://www.prsgroup.com/explore-our-products/icrg/.

[60] Liddle HA (2018). Multidimensional family therapy as a community-based alternative to residential treatment for adolescents with substance use and co-occurring mental health disorders. J. Subst. Abuse Treat..

[61] Westerlund J (2008). Panel cointegration tests of the Fisher effect. J. Appl. Econom..

[62] Chudik A, Pesaran MH (2015). Common correlated effects estimation of heterogeneous dynamic panel data models with weakly exogenous regressors. J. Econom..

[63] Swamy PAVB (1970). Efficient inference in a random coefficient regression model. Econometrica.

[64] Larsson R, Lyhagen J, Löthgren M (2001). Likelihood-based cointegration tests in heterogeneous panels. Economet. J..

[65] Qin L, Kirikkaleli D, Hou Y, Miao X, Tufail M (2021). Carbon neutrality target for G7 economies: Examining the role of environmental policy, green innovation and composite risk index. J. Environ. Manag..

[66] Chudik A, Mohaddes K, Pesaran MH, Raissi M (2016). Long-run effects in large heterogeneous panel data models with cross-sectionally correlated errors. Adv. Econ..

[67] Baltagi BH, Pesaran MH (2007). Heterogeneity and cross section dependence in panel data models: Theory and applications introduction. J. Appl. Economet..

[68] Breusch TS, Pagan AR (1980). The Lagrange multiplier test and its applications to model specification in econometrics. Rev. Econ. Stud..

[69] Pesaran MH (2007). A simple panel unit root test in the presence of cross-section dependence. J. Appl. Economet..

[70] Ahmed Z (2022). How do green energy technology investments, technological innovation, and trade globalization enhance green energy supply and stimulate environmental sustainability in the G7 countries?. Gondwana Res..

